# Oral Health Conditions of Preschool Children Among Birth Cohort Studies: A Scoping Review

**DOI:** 10.3290/j.ohpd.c_1990

**Published:** 2025-06-03

**Authors:** Pierre-Jean Berat, Vincent de Andrade, Nolwenn Regnault, Annabelle Tenenbaum, Sylvie Azogui-Levy

**Affiliations:** a Pierre-Jean Berat Dentist and Hospital Practioner, Université Paris Cité, Faculté de santé, UFR d’odontologie, Department of Pediatric Dentistry; Sorbonne Paris-Nord University, Education and Health Promotion Laboratory (LEPS) (UR 3412); AP-HP – Hôpital Louis Mourier – DMU ESPRIT – Service de médecine bucco-dentaire, France. Idea and hypothesis, study design, literature review, data analysis, wrote the manuscript, approved the final version.; b Vincent de Andrade Research Engineer and Documentalist, Sorbonne Paris-Nord University, Education and Health Promotion Laboratory (LEPS) (UR 3412), Villetaneuse, France. Study design, literature search strategy, approved the final manuscript.; c Nolwenn Regnault Dentist, Université Paris Cité, Faculté de santé, UFR d’odontologie, Department of Dental Public Health, France; Conceptualization, study design, approved the final manuscript.; d; e Annabelle Tenenbaum* Dentist, Lecturer, Hospital Practioner, Université Paris Cité, Faculté de santé, UFR d’odontologie, Department of Dental Public Health; Sorbonne Paris-Nord University, Education and Health Promotion Laboratory (LEPS) (UR 3412); AP-HP Groupe Hospitalier Pitié Salpêtrière, Department of Oral and Dental Medicine, France. Idea and hypothesis, study design, literature review, data analysis, proofread the manuscript, approved the final version.; f Sylvie Azogui-Levy* Dentist, Professor, Hospital Practioner, Université Paris Cité, Faculté de santé, UFR d’odontologie, Department of Dental Public Health; Sorbonne Paris-Nord University, Education and Health Promotion Laboratory (LEPS) (UR 3412); AP-HP Groupe Hospitalier Pitié Salpêtrière, Department of Oral and Dental Medicine, France. Idea and hypothesis, study design, literature review, data analysis, proofread the manuscript proofreading, approved the final version. * Joint last authors.

**Keywords:** birth cohort, oral health, preschool children, scoping review.

## Abstract

**Purpose:**

Oral health is an integral part of health and well-being. Through birth cohorts, it is possible to identify health conditions and pathways of exposure that occur earlier in life. The objective of this study was to identify the data collected by birth cohorts regarding the oral health of children aged 0 to 5 years and to determine the relationship between their dental health status and their environment.

**Materials and Methods:**

Five databases were queried: PubMed, Web of Science, Scopus, Embase, and Dentistry & Oral Sciences Source. Eligible articles presented data on children’s oral health before their 6th birthday, based on birth cohorts. They could be based on clinical, epidemiological, or self-reported oral health data obtained during at least one follow-up between birth and the age of 6 years.

**Results:**

3083 articles were identified in biomedical literature databases. After exclusions for various reasons, 359 abstracts and finally 145 full-length articles were read. A total of 101 articles were included in the analysis. These 101 articles came from 43 cohorts on 5 continents. They were published between October 1980 and January 2022. The most frequently identified theme was early childhood caries and its associated factors. Other themes were also studied: quality of life in relation to oral health, children’s use of dental care, eruption of primary teeth, enamel anomalies, dental trauma, occlusion, and parafunctions in childhood.

**Conclusion:**

Preschool children’s oral health has been widely studied in terms of caries and its risk factors. However, biopsychosocial determinants have to date been insufficiently studied in these birth cohorts.

Oral health is an integral part of health and well-being.^
84
^ It is dynamic and multidimensional.^
84
^ Good oral health enables essential functions such as feeling, eating, swallowing, speaking and smiling.^
56,84
^ Good oral health in children allows harmonious growth of the craniofacial complex and functional balance.^
56
^


Alterations in children’s oral health, most commonly associated with dental caries, can be responsible for worsening child health.^
52,84
^ This can include pain, infection, impaired mastication and early tooth loss.^
84,88,91
^ It can also have psychological or social consequences for the child: fear of smiling, isolation, decreased school performance, school absenteeism. If this deterioration persists, tooth decay can affect the quality of life of the child and family members.^
18,84
^ Children’s oral health differs according to their origins and family socioeconomic status; thus, it is a key marker of social inequality in healthcare that persists throughout life.^
125
^


Fisher-Owens et al^
35
^ proposed a model based on the determinants of children’s oral health. The core of this model contains the main determinants of children’s oral health, focusing on caries. Surrounding them are determinants related to child-level influences, family-level influences, or community-level influences. The model incorporates the 5 key areas of health determinants identified in the literature: genetic and biological factors, social environment, physical environment, health behaviours, as well as medical and dental care. Finally, the model integrates the time component, recognising the evolution of oral diseases such as caries.^
35
^


The evolution of a child’s oral health is related to the life course.^
35,84
^ Oral diseases are widespread and chronic, therefore needing time to develop.^
52,81,91
^ Exposure early in life has long-term consequences for adult health.^
55
^ The social and biological risks accumulated over the life course, particularly during the critical periods of early life, are identified as the main determinants in adult health.^
55,85,100
^


Birth cohort studies suggest that early life experiences influence oral diseases in adulthood.^
83
^ Birth cohort studies also make it possible to study the sequence of risk factors throughout life, which is referred to as life-course epidemiology.^
55
^ Cohort studies make it possible to investigate the successive occurrences of many social and biological processes, the life course of the child, and the child’s development in his/her environment.^
55,113
^ They provide an understanding of natural history and causality of oral diseases and disorders, as well as the salutogenesis actions needed to promote and protect oral health.^
81,86
^ Through following-up cohorts of children since birth, it is possible to identify health conditions and pathways of exposure that occur earlier in life.^
81
^


Not only events related to the eruption of the first teeth and the establishment of oral health behaviours are of interest, but also the critical period from birth to the age of 5. As the WHO stated in 2022, the oral health of the youngest children is a major public health issue at the root of social inequalities in health that will persist throughout their lives.^
125
^


Although the first cohort studies are now more than fifty years old, there is no synthesis that identifies all the results provided by cohorts for the critical period of early life (0 to 5 years).^
29,30,83
^ In order to study the etiology, natural history of oral diseases, social trajectories, and oral health behaviours, analysing the results of cohort reviews is a relevant approach.

The objective of this study was two-fold: a preliminary scoping review of the literature on oral health-related birth-cohort studies was conducted to identify the methodology and types of data collected in these studies;^
81
^ and to identify the data collected by birth cohorts regarding the oral health of children aged 0 to 5 years and determine the relationship between their dental health status and their environment.

## Materials and Methods 

A scoping review was chosen as the preferred approach to describe all the data about oral health in children aged 0 to 5 years provided by birth cohorts. While a systematic review attempts to answer a specific question, a scoping review clarifies the key concepts underlying a field of research and the main sources available.^
3
^ A scoping review summarises them and assesses the literature on the topic.^
108
^


In accordance with the guidelines for scoping reviews, the current research was divided into five steps: 1) identification of the scoping review question, 2) development of the search equation to identify relevant articles, 3) article selection and reading, 4) diagraming of the data, and 5) collation and synthesis of the results.^
3
^


The PRISMA extension guidelines for scoping reviews, which were developed according to published guidance by the EQUATOR (Enhancing the Quality and Transparency of Health Research) Network, were rigorously followed.^
108
^


### Research Strategy

To perform the search, 5 databases were queried: PubMed, Web of Science, Scopus, Embase, and Dentistry & Oral Sciences Source (DOSS). The aim was to identify all articles on birth cohorts which were of interest to oral health. In addition to keyword searches associated with birth cohorts, we included the names of known cohorts in the equation. The names of the cohorts were retrieved from those known by the respective researchers and those listed by the CHICOS project for European cohorts.^
28
^


The initial search equation was designed to query PubMed. It was then adapted to the thesauri of the other biomedical literature database (Appendix 1). The search equations were built and validated with a document researcher. The query equation is as follows:

(“oral hygiene”[Mesh] OR “oral health”[Mesh] OR “oral hygiene”[TW] OR “oral health”[TW] OR “Dental Care for Children”[Mesh] OR “dental car*”[TW] OR “dental health services”[TW] OR “tooth diseases”[Mesh] OR “dental caries”[TW] OR “tooth disease”[TW] OR “tooth dec*”[TW] OR “home dental care”[TW] OR “dental health”[TW])

AND

((“Longitudinal Studies”[Mesh] OR “birth cohort*”[TW] OR “pediatric cohort*”[TW] OR “cohort analys*”[TW] OR “longitudinal stud*”[TW] OR “longitudinal survey*”[TW] OR “longitudinal evaluation*”[TW]) OR “pelotas birth cohort”[TW] OR “flemish environment and health study”[TW] OR “copenhagen child cohort”[TW] OR “danish national birth cohort”[TW] OR “odense child cohort”[TW] OR “northern finland birth cohort”[TW] OR “elfe”[TW] OR “babycare”[TIAB] OR “giniplus”[TW] OR “lisa plus”[TW] OR “rhea cohort” OR “cork baseline birth cohort study”[TW] OR “Lifeways Cross-Generation Cohort Study”[TW] OR “piccolipiu”[TW] OR “kaunas cohort”[TW] OR “ABCD-study cohort”[TW] OR “gecko drenthe”[TW] OR “generation r study”[TW] OR “koala birth cohort study”[TW] OR “mefab”[TW] OR “piama birth cohort”[TW] OR “pride study”[TW] OR “whistler birth cohort”[TW] OR “arcrisk”[TW] OR “norwegian human milk study”[TW] OR “norwegian mother and child cohort”[TW] OR “moba”[TW] OR “krakow cohort”[TW] OR “inma project”[TW] OR “bamse”[TW] OR “inuendo”[TW] OR “avon longitudinal study of parents and children alspac”[TW] OR “born in bradford”[TW] OR “ehl”[TW] OR “growing up in singapore”[TW] OR “millennium cohort”[TW] OR “dunedin multidisciplinary”[TW] OR “SMILE study” [TW] OR “Jena “ [TW] OR “Hong Kong Children” [TW] OR “ PROMISE-EBF study” [TW] OR “IFS”[TW] OR “ Iowa Fluoride Study” [TW])

AND

(“english”[Language]) AND (newborn[Filter] OR allinfant[Filter] OR infant[Filter] OR preschoolchild[Filter] OR “Infant”[Mesh] OR “Child, Preschool”[Mesh] OR “infant*”[TW] OR “preschool*”[TW] OR “newborn*”[TW] OR “toddler*”[TW]).

According to PubMed, TW means Text Words and includes all words and numbers in the title, abstract, other abstract, MeSH terms, MeSH Subheadings, Publication Types, Substance Names, (…), Comment/Correction Notes, and Other Terms.

### Study Selection

Eligible articles were those published up to 12/31/2021, in English only.

An important inclusion criterion was that the publication be the result of research on a birth cohort. Cohort inclusion had to have occurred during pregnancy, at birth or before the first birthday of the child. The children had to be recruited from the general population and not because of a specific medical condition. Multi-year follow-up should also be considered.

Moreover, articles had to focus on children’s oral health before their 6th birthday. They could be based on clinical, epidemiological, or self-reported oral health data obtained during at least one follow-up between birth and the age of 6 years. These results could relate to: oral hygiene habits, dental health, occlusion and parafunctions, children’s use of oral health care, oral health promotion, parents’ involvement in children’s oral health, psychosocial characteristics associated with oral health, and links between general and oral health.

Reviews and conference abstracts were not included. Similarly, interventional studies embedded in cohorts were excluded.

Studies that specifically included premature, low-birth-weight or high-birth-weight children, or populations with other specific characteristics, such as cohorts of adolescents, cohorts of children with a specific medical condition or children exclusively followed by dental services, were not included in this scoping review. Articles studying only permanent teeth, or immunological or oral microbiological analyses, were likewise not included in the scoping review.

Articles identified in the electronic search were imported to the bibliographic software rayan.ai. After article identification, duplicates were removed using rayan.ai software. Titles were first screened independently by at least 2 reviewers. The relevant abstracts were screened with the same protocol. Full texts of relevant articles were then retrieved and examined for suitability. Any disagreements regarding the selection of studies were resolved through discussion with a third reviewer.

### Data Analysis 

Selected articles were read in their full length. The data obtained for all selected articles were processed qualitatively and classified according to the cohort to which they belonged.

The selected articles were summarised according to the number of participants included in the study, the variables studied (sociodemographic, oral health, general health and psychosocial), the descriptive and analytical results reported, and the conclusions.

## Results

**Fig 1 fig1:**
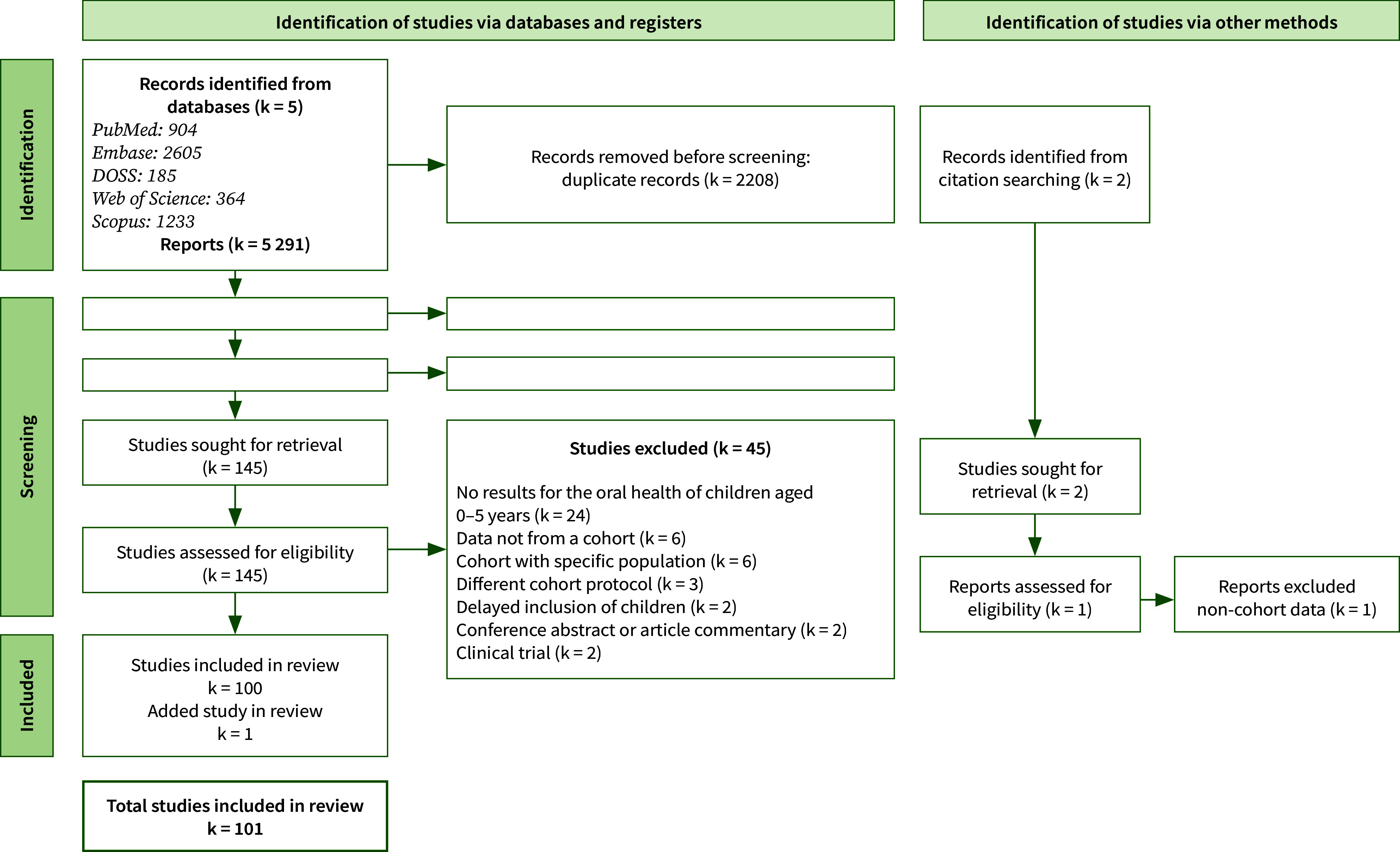
Flow diagram for scoping review.

The flowchart describing article selection is presented in Fig 1. From the 5 biomedical literature databases, 5291 articles were retrieved. After deleting duplicates, we read 3083 titles, then 359 abstracts, and finally 145 articles in their full length. Related searches by reading bibliographic references led to reading a further 2 articles. Finally, 45 articles were excluded. Among these, 24 presented no specific data relating to the oral health of children aged 0 to 5. A further 6 articles were excluded, as they were drawn from a cohort of Brazilian teenage mothers, thus constituting a specific population recruitment. A total of 101 articles were included in the analysis. These 101 articles come from 43 cohorts on 5 continents (Table 1) and were published between October 1980 and January 2022. The main results are presented by cohort in Table 2.

**Table 1 table1:** Publications by cohort

Continent	Number of articles – cohort name (city or district, country), birth cohort start year, references
Africa	2 – PROMISE-EBF (Mbale District,Uganda) , 2006 – 2011 8,9 1 – Birth-to-Ten study – BTT (Soweto – Johannesburg, South Africa), 1990 64
North America	23 – Iowa Fluoride Study – IFS (Iowa, USA), 1992– 1995 19,20,37,44,45,47,48,58–63,67–71,92,96,97,119,120 1 – Carolina Oral Health Literacy project – COHL (North Carolina, USA) 2 2 – Northern Plains Tribal Community (Iowa, USA), 2009 117,118 1 – Iowa (USA), 1992-1994 116 1 – Iowa Medicaid (USA), 2000 23 1 – Quebec (Canada), 2006-2016 4
South America	5 – Ten Steps towards a Healthy Diet for Children Younger than Two Years of Age Implementation Project (Porto Allegre, Brazil) 11,16,17,31,33 8 – Pelotas 2004 (Brazil) 10,14,36,77,80,82,95,107 1 – Pelotas 2015 (Brazil) 94 3 – João Pessoa (state in Northeast Brazil, Brazil) 22,76,104 2 – São Leopoldo (Brazil) 32,33 2 – The Impact of Care in the Practice of Maternal Breastfeeding and Oral Health on the Mother-Child Binome (Sao Paulo, Brazil), 2006 72,73
Asia	4 – Growing Up in Singapore Towards healthy Outcomes – GUSTO (Singapore), 2009-2010 49,110–112 4 – Prospective Cohort Study of Thai Children – PCTC (Thailand), 2000 74,78,79,105 2 – Osaka Maternal and Child Health Study – OCMHS (Osaka, Japan), 2001-2003 102,103 1 – Japan Environment and Children’s Study – JECS (Japan) 109 1 – Born in Guangzhou Cohort Study – BIGCS (Guangzhou , China), 2012-2018 101 1 - Xinhua (China), 2008 127 1 – Kerman (Iran) 87
Europe	2 – Avon Longitudinal Study of Parents and Children – ALSPAC (Avon South West England), 1992 27,54 1 – Surrey (England), 1995 41 1 – Southampton Women’s Survey – SWS (Southampton , England), 1998 – 2002 75 1 – Dundee Study ( Dundee, Scotland), 1993-1994 7 1 – Flandres (Belgium), 2003 57 2 – Jena birth cohort (Jena, Thuringia, Germany), 2010 114,115 1 – Danish health registers (Denmark) 24 1 – Registry-based cohort study (Stockholm, Sweden) 99 1 – Halland Health and Growth study (Halland, Sweden), 2007 – 2008 13 1 – Sweden 42 1 – (Lohja, Finland), 1983-1985 1 4 – Norwegian Mother and Child Cohort Study – MOBA (Akershus Count, Norway) 2002 121–124
Oceania	2 – The Dunedin Multidisciplinary Child Development Study (Dunedin, New Zealand), 1972 29,30 2 – The Pacific Islands Families – PIF (Auckland , New Zealand) 89,90 1 – Growing Up in New Zealand (Auckland, Manukau & Waikato, New Zealand) 2009 – 2010 106 1 – Christchurch Child Development Study (Christchurch, New Zealand), 1977 5 3 – Study of Mothers’ and Infants’ Life Events affecting oral health – Smile (Adelaide, Australia), 2013-2014 6,25,46 1 – (South West Queensland, Australia) 50 1 – South Australian Aboriginal Birth Cohort – SAABC (South Australia, Australia), 2011 51 4 – The VicGeneration – VicGen (Victoria, Australia), 2008 15,21,39,40 1 – Healthy Smiles Healthy Kids – HSHK (Sydney, Australia), 2009 65 1 – Gudaga (New South Wales Australia), 2005-2007 38


**Table 2 Table2:** Summary of results by cohort

Reference number of the studies selected	Cohort Name (City or District, Country)	Number of children included	Key results
8,9	PROMISE-EBF (Mbale District, Uganda)	765 mother-child dyads	Relationship between exclusive breastfeeding and dental caries to be adjusted for confounding factors (socioeconomic status, child nutrition and oral hygiene). Exclusive breastfeeding for 24 weeks → protective effect on early childhood caries (ECC) Association between ECC and caregiver’s caries experience or OHRQoL in a low-income country
64	Birth-to-Ten study – BTT (Soweto – Johannesburg, South Africa)	2000 children	Any relationship between incidence of tooth decay and average nutrient consumption
19,20,37, 44,45,47, 48,58–63,67–71, 92,96, 97,119, 120	Iowa Fluoride Study -IFS (Iowa, USA)	1390 children	Protective factors against caries: breastfeeding until 6 months, fluoride intake, or more regular brushing. Brushing frequency: positively associated with mother’s and father’s level of education. Risk factors for tooth decay: exposure to tobacco, low level of parental education young parents at child’s birth mother with high BMI or tooth decay, low birth weight, high consumption of snacks and sugary drinks, eating larger quantities at mealtimes, low fluoride content in water (if breastfed for less than 6 months), presence of enamel hypoplasia. No relationship between socioeconomic status and tooth decay. Period at risk of fluorosis of deciduous teeth: fluoride intake between 6 and 9 months (mainly involving deciduous second molars). Fluoride intake increases with: child’s age (stable intake from age 3), mother’s low level of education, or low household income. Use of dental services before age 3 encouraged by: high level of maternal education and higher or lower household income.
116	Iowa (USA)	700 children	Role of parafunctions (pacifier use, digital sucking) on occlusal characteristics and arch parameters, no role of breastfeeding duration. Persistence of malocclusions after cessation of digital or pacifier sucking.
23	Iowa Medicaid (USA)	6322 children	No relationship between time of first visit and frequency of infant well-child visits. Earlier first preventive dental visit associated with: mother’s non-white background, mother’s preventive dental check-up 12 months before the child’s birth, numerous preventive medical visits between the ages of 1 and 3, preventive medical visits to various professionals
2	Carolina Oral Health Literacy /	119 children	Relationship between evolution of mother’s dental neglect of child and evolution of mother’s oral health literacy. Information about child’s oral health during pregnancy and mother’s intention to change oral hygiene habits towards favourable behaviour → positive behaviour towards child’s oral health.
117,118	Children living in Northern Plains Tribal Community (USA)	244 children	Tooth decay on incisors at 3 years of age. Risk factors for tooth decay: younger maternal age, higher maternal DMFT (decayed, missing and filled tooth), more people living in the household, or higher consumption of sugary drinks. Maternal DMFT related to child’s dmft.
4	(Quebec, Canada)	790 758 children	Prenatal use of psychoactive substances (alcohol, drugs, etc.): ECC risk factor (more significant for alcohol).
11,16,17,31,34	Ten Steps towards a Healthy Diet for Children Younger than Two Years of Age Implementation Project (Porto Alegre), Brazil	715 mothers	Relationship between ECC and breastfeeding over 24 months or breastfeeding on demand, or exposure to a high proportion of cariogenic foods and beverages. Higher risk of dental trauma in children with smaller head circumference at birth, overweight or obese at 12 months, or boys Child’s dental visit encouraged by mothers with a high level of education or families with low socio-economic status. No association between child’s dental visit and size or composition of the oral health team, or caries experience or history of dental trauma. Higher perceived impact on quality of life → child’s dental visit.
10,14,36, 77,80,82, 95,107	Pelotas 2004	4231 children	Exclusive breastfeeding protective factor for open bite and severe malocclusion at 5 years (protective effect cancelled out by pacifier sucking). Breastfeeding beyond 24 months → ECC. Mothers’ oral health predictive of their children’s oral health. Prevalence of dental pain related to: socioeconomic status, mother’s oral health litteracy, or children’s caries experience at age 5. Oral mucosal lesions → negative impact on oral health quality of life. Risk factors for dental fear: children who have never had a dental visit, children with tooth decay or dental pain (independent of socio-economic status and maternal characteristics), mothers with a low level of education, low family income. Child’s dental visits more frequent due to high level of maternal education and socioeconomic status, mothers over 30 years of age, mothers regularly use dental services, mothers having benefited from preventive information, children helped to brush their teeth, no children’s dental fear.
94	Pelotas 2015	4014 children	A lower mean number of teeth was observed in children from non-white mothers, early preterm children, and shorter children at birth and at 12 months. A higher number of teeth was observed for mothers with excessive weight gain during pregnancy, mothers who smoked during pregnancy, weightier children at birth and at 12 months, and for children with larger heads at birth and at 12 months.
22,76,104	(João Pessoa), Brazil	228 children	Small for gestational age → enamel defects. No relationship between enamel defect and gender. Risk factors for tooth decay: enamel defects (opacities or hypoplasias), night-time breastfeeding at 18 months, night-time bottle-feeding, or absence of daily brushing or use of fluoride toothpaste.
32,33	The Impact of Care in the Practice of Maternal Breastfeeding and Oral Health on the Mother-Child Binome (São Leopoldo), Brazil	500 children	Low maternal education is a risk factor for cariogenic feeding practices → possible mechanisms for the relationship between low socio-economic status and tooth decay. No relationship between cariogenic feeding practices and household income, anthropometric and demographic data. Risk of traumatic dental injuries was significantly greater given: higher household income, a greater height for age, greater number of teeth erupted at 12 months of age.
72,73	The Impact of Care in the Practice of Maternal Breastfeeding and Oral Health on the Mother-Child Binome (São Paulo), Brazil	80 mother-child dyads	Relationship between neglect of oral hygiene and the development of tooth decay. Risk factors for malocclusion: sucking habits, low rates of breastfeeding, and nocturnal mouth breathing. Finger sucking habit or low rates of breastfeeding → overjet and open bite. Pacifier sucking habit → overjet, open bite and later overbite. Bottle feeding or nocturnal mouth breathers → posterior crossbite.
49,110–112	Growing Up in Singapore Towards healthy Outcomes – GUSTO (Singapore)	1237 children	Early eruption of primary teeth associated with: infant weight gain in the first 3 months, ethnic origin or high maternal age at birth. Presence of pain or fever during eruption of primary teeth associated with: exposure to tobacco smoke during pregnancy, ethnic origin or Caesarean section Prenatal plasma vitamin D deficiency seems protective. Increased incidence of dental caries at age 2 associated with mother’s ethnic origin, high maternal age (34 and over), night-time breastfeeding in the first weeks of life, child’s higher BMI, non-compliance with dietary recommendations, children’s dental visits before 24 months old, high plaque index, toothbrushing ≥ 1 / day or use of fluoride toothpaste A history of dental consultations linked to the presence of an ECC: this would reflect the use of dental services for curative and not preventive treatment
79,78,74,105	Prospective Cohort Study of Thai Children – PCTC (Thailand)	860 children	Buccal surface of the maxillary incisors was the most affected before the age of 18 months. Factors associated with ECC: socio-environmental factors (rainwater or well water, mother’s no schooling, male child), or behavioural factors (sleeping with bottle at 30 months, prolonged breastfeeding beyond 12 months (often associated with night-time breastfeeding and frequent snacking) Breastfeeding between 6 and 12 months: protective factor against ECC
102,103	Osaka Maternal and Child Health Study – OCMHS (Osaka, Japan)	867 children	Child’s dental visit for 40% of children in Japan Protective factor against ECC: high parental education levels (especially maternal education) ECC risk factor: prolonged breastfeeding, use of bottles with sweetened liquids other than milk or introduction of solid foods at 6 months of age or later No relationship between maternal occupation or household income and the risk of children’s dental caries
109	Japan Environment and Children’s Study – JECS (Japan)	83,954 children	Significant association between maternal bond disorders and lower frequency of child toothbrushing Increased risk of ECC and lower frequency of child toothbrushing
101	Born in Guangzhou Cohort Study – BIGCS (Guangzhou , China)	476 children	Possible link between ECC and mothers considered likely to suffer from depression
127	Xinhua (China)	225 children	Children are more likely to develop ECC given: low maternal education, high family income, children shorter than average, enamel hypoplasia or visible dental plaque.
87	Kerman (Iran)	117 children	No relationship between ECC and mode of delivery or child’s ABO blood groups Risk factors for ECC: poor maternal oral hygiene or higher maternal mutans streptococci
27,54	Avon Longitudinal Study of Parents and Children – ALSPAC (Avon South West England)	1314 children	Relationship between high increased risk of caries at age 5 and low maternal education, living in council or housing association, or breastfeeding duration Persistent non-nutritive sucking leads to malocclusion
41	Surrey (England)	2300 children	Positive relation between visible dental plaque and the mean total eating / drinking episodes regardless of toothbrushing frequency.
75	Southampton Women’s Survey – SWS (Southampton, England)	3159 children	Relationship between eruption of deciduous teeth and maternal socioeconomic status, maternal smoking and reduced physical activity of the mother during pregnancy
7	Dundee Study (Dundee, Scotland)	1455 children	High number of decayed, missing and filled primary tooth (dfmt) associated with unemployed parents, low birth weight or maternal smoking
57	Flandres (Belgium)	1057 children	dfmt increase with: age, sibling rank, tobacco exposure, screen time over 1 hour per day, presence of cavities at age 3, lower education level of father and mother. Protective factor against dental caries: parental brushing assistance
114,115	The impact of a preventive program on the oral health of Thuringian children in (Jena, Thuringia, Germany)	512 children	No association between enamel defect and tooth decay Significant association between enamel defects and: prematurity, disease or disability, medication during the first year of life, hospitalisation. Malocclusion significantly associated with non-nutritive sucking
24	Danish health registers (Denmark)	883,380 children	Parents with severe mental illness → no preventive dental visits for the child between 0 and 5 years of age, and presence of tooth decay at age 5
99	Registry-based cohort study (Stockholm, Sweden)	74,748 children	Relationship between low gestational age and tooth decay at 3 years if no smoking during pregnancy
13	Endocrine cohort issue en partie de Halland Health and Growth study (Halland, Sweden)	551 children	Increased dfmt given: preterm infants, low birth weight/height and infant blood glucose U-shaped relationship between dfmt and child weight
42	Sweden	212 children	Relationship between eruptions (early or delayed) of primary incisors and primary molars, or primary and permanent teeth
1	(Lohja, Finland)	54 children	Increased incidence of tooth decay: if juice or refined sugar products are snacked on, or in absence of the use of fluoridated products Inverse relationship between frequent close salivary contact between mother and child, associated with SM infection and the incidence of tooth decay in deciduous dentition
121–124	Norwegian Mother and Child Cohort Study – MOBA (Akershus Count, Norway)	1607 children	ECC development associated with tooth-brushing habits and sugar consumption, regardless of household characteristics Toothbrushing frequency at 1.5 years = toothbrushing frequency at 5 years High dfmt at age 5 associated with episodic brushing, non-compliance with the recommendations for sugar and fat consumption (including drinks at night), low level of maternal education, an overweight, asthmatic, allergic or non-Western mother, change in family situation (from 2 to 1 parent)
29,30	The Dunedin Multidisciplinary Child Development Study (Dunedin, New Zealand)	923 children	Dental erosion can be identified from 48 months. Dental erosion associated with bottle feeding at 36 months. Parents’ use of school dental services is related to their perception of the benefits of these services. Relationship between parents’ oral health and that of their children, in terms of dfmt and oral hygiene habits Parents’ assessment of their child’s oral health is related to the child’s dfmt. dfmt decreases with age of first dental visit
89,90	PIF – Pacific Islands Families study (Auckland , New Zealand)	1398 children	Children with mothers more aligned traditional cultural orientation have greater unmet needs The acculturation level of the mother in the country of residence has a minor effect on the child’s dfmt. Relationship between acculturation groups for children’s toothbrushing frequency and school dental service enrollments Restorative treatments and extractions associated with: prolonged breastfeeding (over 1 year) → delayed introduction of solid foods mother’s toothbrushing ≤ 1/day child snacking or drinking prior to bed, mother’s Pacific island origin.
106	Growing-up in New Zealand (Auckland, Manukau & Waikato, New Zealand)	6853 children	Dental caries associated with: toothbrushing frequency, parental help with brushing, duration breastfeeding, child snacks or drinks prior to bed, live in socio-economically deprived areas or according to the type of food eaten.
5	Christchurch Child Development Study (Christchurch, New Zealand)	1265 children	No dental visit before age 4 for 16.7% of children.Relationship between rates of utilization of dental care services and family social background or quality of child care. Relationship between early dental care and other aspects of early child care.
6,25,46	Study of Mothers’ and Infants’ Life Events affecting oral health – Smile (Adelaide, Australia)	2181 children	ECC associated with: free sugars intakes, living in a disadvantaged environment, or mother’s caries experience (effect attenuated by mother’s socioeconomic status and her oral hygiene habits).
50	(South West Queensland, Australia)	1017 children	Dental erosion in primary dentition increased with age.
51	South Australian Aboriginal Birth Cohort – SAABC (South Australia, Australia)	448 children	Clinically detectable tooth decay between 24 and 36 months. Mother’s experiences of racism impact on both toothbrushing and toothache.
15,21,39,40	The VicGeneration – VicGen (Victoria, Australia)	467 children	Oral health problem, other than dental caries (dermatological lesions of the mucosa, tongue-tie, ..) for around 8% of children.Child’s dental visit for 45% of children before the age of 5 years. No relationship between dfmt and frequency of child’s dental visits. Risk factors for ECC: juice and soda consumption, bacterial load, exposure to cigarette smoke or suboptimal exposure to water fluoridation.
65	Healthy Smiles Healthy Kids – HSHK (Sydney, Australia)	1035 children	ECC more frequent if: breastfeeding duration over 1 year or low socioeconomic status. No relationship between the child’s dietary trajectory and ECC.
38	Gudaga (New South Wales Australia)	149 children	Parents’ concerns about their child’s oral health increase over the years. Aboriginal children: few dental visits between the ages of 2 and 5, despite a high caries index. Possible mismatch between parents’ concern about their child’s perceived oral health and their child’s oral disease.
			

In this scoping review, the most frequently identified theme is early childhood caries (ECC) and its associated factors. These frequently studied factors include: family sociodemographic factors, parents’ health and use of psychoactive substances, the child’s health, breastfeeding and the child’s eating habits, the oral hygiene of the child and parents, and the role of fluoride. Other themes were also studied thanks to birth cohorts: quality of life in relation to oral health, children’s use of dental care, eruption of primary teeth, enamel anomalies, oral trauma, occlusion and parafunctions in childhood.

### Early Childhood Caries

#### Sociodemographic factors

Several sociodemographic factors have been identified as indicators of early childhood caries. Children with caries experience have younger parents.^
69
^ The mother’s age at birth appears to be a means of identifying children at greater risk of developing caries.^
39
^ According to some studies, children whose mothers are younger are more likely to have ECC.^
54,117
^ For other birthcohorts, children whose mothers are older are more likely to have ECC.^
112
^


Furthermore, not being the first child is more frequently associated with the presence of caries.^
57
^ Variation in the number of people living in the household,^
121,123,124
^ or a greater number of people living in the household^
117
^ are characteristics found in children with ECC. Parental origin seems to play a role. Parents of non-Western origin or with a culture different from that of the country in which the family resides have children with more caries.^
89,90,112,121,123,124
^


Parental acculturation level is identified as a factor associated with caries. Low parental education is associated with greater caries experience in children.^
48,57,68,69,78,79,127
^ A higher level of parental education, particularly maternal education, is significantly associated with a reduced risk of caries in Japanese children.^
103
^


Children whose parents are unemployed,^
7
^ or whose family’s socioeconomic status is considered low, present caries more frequently.^
19,20,25,39,62,65,68,69
^ But no association was established in a Japanese cohort between the mother’s occupation or household income and the risk of childhood caries.^
103
^ Children in developing countries whose families are considered to have a high socioeconomic status have more experience of tooth decay than other children.^
127
^


Lastly, children exposed to monitors/screens for more than 1 hour a day have more carious lesions.^
57
^


#### Feeding practices

A low educational level of mothers is a risk factor for cariogenic dietary practices in the first year of life, and represents one of the possible mechanisms by which lower socioeconomic status is associated with the presence of caries.^
33
^ Cariogenic feeding practices are not correlated with family income, demographic characteristics, or the child’s health at birth.^
33
^ Similarly, according to the Iowa Fluoride Study cohort, the link between socioeconomic status and the presence of tooth decay may be due to different feeding practices.^
45
^ Children from low socioeconomic status families consumed more fruit juice or soda than those from high socioeconomic status families. However, no statistically significant difference was found when comparing toothbrushing frequency or use of fluoride toothpaste between these two groups.^
45
^ For the SMILE cohort, the child’s eating habits seem to be associated with the mother’s sociodemographic characteristics.^
46
^


These results suggest that snacking and drinking habits are a key difference between families of high and low socioeconomic status, and could partly explain the differences in caries experience between subjects of different socioeconomic status.^20,33,45,48,62,67–70^


A higher consumption of sugar, especially refined sugar (from sweetened beverages such as sodas or fruit juices), and fat than recommended, as well as a high intake of snacks and nighttime bottle-feeding are modifiable risk factors for caries.^1,16,20,34,39,40,46,48,62,67–70,76,78,79,89,102,104,106,112,117,121,123,124^


Feeding practices may be associated with the mother’s sociodemographic factors, the child’s early feeding experiences, and the childcare arrangements.^6,15,20,48,62,67–70^ Children with parent care only tend to eat more optional foods (snack, soda..) than children with formal childcare. This difference is not observed in the caries index or frequency of dental visits among children aged 0 to 5 years.^
15
^


No relationship was found between average nutrient intake and the incidence of tooth decay.^
64
^


Children who brush their teeth infrequently at 1.5 years of age consume sugar more often at the same age, and have more caries at age 5.^
124
^


#### Breastfeeding

The link between exclusive breastfeeding and caries needs to be adjusted for confounding factors such as family sugar intake, duration of breastfeeding, anthropometric status, maternal education, household income, and family oral hygiene. Marital status has a second-order effect on this relationship.^
8
^ Breastfeeding for up to 6 months appears to be a protective factor against ECC.^
8,19,48,62,68,70,74
^ Breastfeeding also appears to be a protective factor against caries between 6 and 12 months of age.^
74
^ Breastfeeding beyond 12 months is often associated with nocturnal breastfeeding and frequent snacking.^
74,76
^ The relationship between breastfeeding and tooth decay is uncertain (due to associated risk factors). Prolonged breastfeeding beyond 12 months appears to increase the risk of carious lesions.^
65,74,89
^ Prolonged breastfeeding beyond 18 or 24 months, or breastfeeding on demand during the day, are also risk factors for developing a severe form of ECC.^
16,17,34,76,82,102,106
^


Finally, on-demand breastfeeding during the day, or low fluoride levels in water if the child has been breastfed for less than 6 months, are also risk factors for caries in children.^
17,48
^


#### Oral hygiene

The protective factors against caries reported by the cohorts are fluoride intake and more frequent toothbrushing.^19,20,48,62,67–70,109,119^ On the other hand, a lower frequency of toothbrushing or lack of use of fluoride toothpaste appear as risk factors for developing caries in pre-school children.^
1,19,20,37,62,73,76,104,106,109
^ Suboptimal exposure to water fluoridation through consumption of rainwater or well water appears to be a risk factor for developing carious lesions on deciduous teeth.^
39,78,79
^


The presence of plaque is associated with the presence of tooth decay.^
57,112,127
^


Brushing frequency was positively associated with both mother’s and father’s level of education.^
37,44,58,61,63
^ Parental help with toothbrushing seems to be a protective factor against tooth decay.^
57,106
^ The age of beginning brushing does not seem to affect the risk of caries.^
57
^


When children were 2 years old, almost all of them used fluoride toothpaste to brush their teeth.^
37
^ Brushing frequency at 1.5 years of age remains the same until 5 years of age. Children who rarely brush their teeth at 1.5 years old consume sugar more often at the same age and have more cavities at the age of 5 years old.^
122
^ Fluoride intake increases with child’s age, mother’s low level of education, and low family income.^
61
^ From age 3, fluoride intake seems stable.^
61
^


By age 5, around 40% of children were brushing their teeth twice a day, as recommended.^
37
^ The presence of an older sibling or the parents’ Western background favours twice-daily brushing.^
89,122
^ In some studies, toothbrushing frequency did not appear to modify the risk of caries,^
57
^ while in others, it was a risk factor for caries disease.^
106
^ Regardless of brushing frequency, plaque index remains high in the presence of frequent snacking.^
42
^ Irrespective of family characteristics, brushing and sugar consumption play a role in the early development of caries.^
121,123,124
^


Children aged 0 to 5 who brush their teeth twice a day have more positive attitudes towards their oral health.^
122
^ A positive attitude towards the child’s oral health is established at the child’s birth if the mother has received information on this subject during pregnancy and intends to adopt oral hygiene habits favourable to the child’s oral health.^
2
^


Maternal bonding disorders lead to lower frequency of toothbrushing in children.^
109
^ Mothers’ experiences of racism have a negative impact on children’s toothbrushing.^
51
^ History of dental pain was more frequently associated with mothers’ experiences of racism.^
51
^


Mothers play the most important role in their children’s home-care habits.^
37,44,58,61,63
^ There is a relationship between mothers’ and children’s brushing behaviour before bedtime.^
46
^ Younger mothers dispense more toothpaste onto their children’s toothbrushes.^
37
^


#### Child health

Being a boy seems to be an indicator of caries disease in a Thai cohort.^
78,79
^ Children’s median age-adjusted weight, height, and BMI did not differ according to caries experience.^
68
^ Low socioeconomic status seems to play a key role in the development of caries, independent of the role played by obesity.^
68
^ There appears to be a U-shaped relationship between caries and weight: children with the lowest weights and those who are overweight have more decayed teeth.^
13,112
^ A study of a Chinese cohort revealed that children aged 0 to 2 years old with shorter-than-average heights are at greater risk of developing tooth decay.^
127
^


Children born moderately preterm or at late preterm have statistically significantly more caries lesions than full-term babies, especially if the mother did not smoke during pregnancy.^
13,99
^ Children born small or underweight for their gestational age have more tooth decay.^
7,13,48,57
^


There is a relationship between normal and fasting blood glucose levels in children and the presence of decayed or filled primary teeth.^
13
^


There is an inverse association between frequent close salivary contact between mother and infant associated with mutans streptococci infection and the incidence of caries in primary dentition.^
1
^


#### Parental health and oral hygiene

Parents’ oral health is a factor associated with their children’s oral health.^
10,28
^ Children whose parents (most often the mother) do not brush their teeth at the child’s birth or have poor oral hygiene, have a high presence of maternal mutans streptococci and statistically significantly more tooth decay.^
57,87,89
^ Maternal caries experience was associated with a higher prevalence of childhood tooth decay.^
9,45,117,121,123,124
^ The effect is modulated by the mother’s socioeconomic status and her oral hygiene habits.^
45
^


Children born to parents suffering from schizophrenia, bipolar disorder, or depression are at increased risk of developing caries by age 5.^
24,101
^ Prenatal exposure to psychoactive substances (alcohol, drugs, etc.) seems to be a risk factor for caries in childhood. This trend was more significant for alcohol than for other substances.^
4
^ Children whose mothers are obese, have asthma or allergies have more carious lesions on their primary teeth.^
68,121,123,124
^ No relationship was identified between ECC and mode of delivery or the mother’s ABO blood group.^
87
^


#### Quality of life related to oral health

Early childhood caries (ECC) is associated with the caregiver’s caries experience. Children with caries experience have an altered quality of life for themselves and their families in relation to their oral health.^
9
^ A lower number of decayed, missing, and filled primary teeth (dft) was observed in children aged 0 to 5 with parents who rated their own teeth as “average” compared to children whose parents rated their teeth as less than “average”. The same trends were observed in the assessment of oral hygiene.^
29
^


There is a relationship between parents’ qualitative assessment of their children’s oral health and the child’s dfmt index.^
29
^ Caregivers with caries were more likely to perceive quality-of-life deficits in their children and families. Conversely, caregivers who perceived their child’s oral health as good were less likely to perceive quality-of-life deficits in children and families.^
9
^


Parents’ concerns about their child’s oral health increase over time. As a result, there may be a disconnect between parents’ concern about their child’s perceived oral health (and use of dental care) and their child’s needs.^
38
^ Parents also seem well informed about their children’s dental treatment needs.^
28
^


Dental pain is more frequently associated with tooth decay.^
10
^ Socioeconomic status, mothers’ oral health literacy and children’s caries experience at age 5 were associated with dental pain in primary teeth.^
10
^ The prevalence of dental pain was reduced in children aged 0 to 5 whose mothers were able to control the children’s oral hygiene habits.^
10
^ Oral mucosal lesions have a negative impact on oral health quality of life.^
77
^


### Use of Dental Care

Dental visits increase with child’s age,^
45
^ and early dental visits reduce the dfmt index.^
29
^ Early dental visits (between 9 and 24 months) are associated with the presence of severe ECC.^
112
^


Between 40% and 85% of children have visited the dentist before the age of 5.^
5,21,103
^ Two percent (2%) of children have had a first dental visit at age one, 11% at age two and 31% at age three. Almost all children who have not had a dental visit by age 3 have had one by age 6.^
59
^ 19% of children who had a dental visit before the age of 3 benefited from professional fluoride application.^
96
^


Children whose mothers had a high level of education, and whose family income was high or low, were more likely to have had a dental visit before age 3, depending on country.^
31,96
^ Children from high socioeconomic status families are more likely to have had a dental visit in the first 5 years of life than children from low socioeconomic status families.^
31,45
^


A mother over 30 years of age at birth, regular oral health follow-up by the mother, a preventive dental visit by the mother 12 months before the child’s birth, and the fact that the child has been brought for consultation by different providers during preventive visits are all factors associated with the earliness of a first dental visit.^
14,23
^ The time of the first dental visit was not associated with the frequency of the infant’s visits to the GP.^
23
^ Perceived higher impact on quality of life, and the mother receiving preventive oral health information were positively associated with the child having an earlier dental visit.^
14,31
^


Western background favours use of school dentist services regardless of severity of caries disease.^
38,89,90
^ Use of school dental services is related to parents’ perception of their benefits.^
29
^


In the absence of dental fear, children aged 0 to 5 attended dental services as preventive care in Brazil.^
14
^ In Brazil, children aged 0 to 5 who did not consult the dentist or only in an emergency were are children who had dental fear.^
107
^


Non-use of dental services was associated with family income, maternal ethnic status, non-attendance at Community Health Nurse, preschool education.^
5
^


### Eruption of Primary Teeth

Various events during pregnancy influenced the timing of eruption of primary teeth.^
75,94,110,111
^ Aside from genetic factors, reduced physical activity during pregnancy, infant weight gain, premature birth, birth size, child sex, larger head circumference, maternal smoking, ethnic origin, socioeconomic status and mother’s age at birth also influence the age of eruption of primary teeth.^
75,94,110,111
^ When primary incisors erupt early, so do primary molars. Similarly, if primary teeth erupt early, permanent teeth also erupt early.^
43
^


Tobacco smoke exposure during pregnancy, ethnic origin, and Caesarean section are risk factors for pain or fever during the eruption of primary teeth. Prenatal plasma vitamin D deficiency seems to reduce this risk.^
110,111
^


### Structural Defects in Primary Teeth

Enamel defects in primary teeth are statistically significantly associated with prematurity, the presence of systemic disease or disability, medication taken during the first year of life, or hospitalisation.^
114
^ They also seem to be associated with intrauterine nutrient deficiencies.^
22
^ Gender has no effect on the presence of enamel defects.^
22
^


For some authors, primary tooth defects such as opacities or hypoplasias are risk factors for childhood tooth decay,^
48,76,104,127
^ although this relationship is not always statistically significant.^
114
^


Black stains seem to be related to reduced dental tooth decay, although not statistically significantly so.^
36
^


According to the Iowa Fluoride Study cohort, fluorosis of primary teeth appears to be mainly the effect of postnatal fluoride exposure. This fluoride intake is mainly via fluoridated drinking water used to reconstitute infant food, and sometimes via toothpaste. Fluoride intake increases with the child’s age, mother’s low level of education, and low family income. There appears to be no link between fluorosis and fluoride concentration in toothpaste. Increased fluoride intake between 6 and 9 months of age appears to greatly increase the risk period for developing fluorosis of primary teeth (mainly affecting primary 2nd molars).^
47,58,60,71,97,120
^


### Occlusion and Sucking Habits in Children Aged 0 to 5

Children’s parafunctions, such as sucking on pacifiers, non-nutritive sucking or mouth-breathing, were significantly predisposed to malocclusions in deciduous dentition.^
27,72,80,115,116
^


Children with a pacifier-sucking habit had a higher risk of overjet, anterior open bite or overbite, and a lower risk of posterior crossbite.^
72,116
^ Children who sucked their fingers, or those who with low rates of breastfeeding, were more likely to have an overjet and open bite.^
72,116
^ Finger sucking is related to increased palate depth and decreased width of the dental arch.^
116
^ Malocclusions persist well beyond the cessation of the pacifier or digit habit, but they reduce spontaneously.^
27,116
^


Sucking habits lead to an increase in posterior crossbite.^
27,72
^ In the absence of sucking habits, the duration of breastfeeding does not seem to influence dental arch and occlusal characteristics.^
116
^ Exclusive breastfeeding is a protective factor against open bite and severe malocclusion in children aged 5. This protective effect of breastfeeding is negated by sucking on pacifiers.^
80
^


### Traumatology of Primary Teeth

The risk of dental trauma was higher among children with a small head circumference at birth, children who were overweight or obese at 12 months of age, boys, taller children, or those with more teeth erupted at 12 months of age.^
11,32
^ The analysis revealed that socioeconomic variables and other height and weight variables were not statistically significantly associated with the risk of trauma to primary teeth.^
11
^


## Discussion

This scoping review identified 43 cohorts in 20 countries spread over 5 continents, with different levels of development, wealth, and healthcare systems. We chose to include articles focusing on infants and pre-school children, i.e., articles providing data on oral health in the 0-5 age group, rather than all children. Some cohorts were excluded from our analysis, as they only provided data on oral health from the age of 6. This review was exclusively concerned with oral health data about children aged 0 to 5, in addition to data concerning the child’s environment. It should be noted that the present study did not include data from complementary, biological, or microbiological examinations, despite these being included in the Fisher-Owens model.^
35
^ We also excluded from our analysis all articles studying microbiological or immunological parameters in these children.^
81
^ This scoping review has identified other articles than did Peres et al.^
81
^


Birth cohorts tend to be tools for investigating the origin of oral health impairment rather than questioning what makes it possible to maintain good oral health.^
86
^ At the end of this scoping review, it is apparent that oral health has been very widely studied through caries disease, a phenomenon also observed by Peres et al.^
81
^


The various protective and risk factors for caries disease identified, as well as data on diet and oral hygiene, corroborate the Fisher-Owens model in several aspects.^
35
^ Birth cohort studies aim to identify biological or environmental risk factors for diseases and, in our case, for the oral health of children aged 0 to 5.^
113
^ Socioeconomic determinants such as household income and parents’ level of education are often studied to define groups of children at high risk of caries. They have a limited mediating effect. They are most often associated with other covariates, in particular dietary ones, and it is this link – non-compliance with dietary recommendations, difficulties in accessing care and caries – which explains why families with disadvantaged sociodemographic profiles present a deterioration in oral health.^
31,41,45,96
^


Insufficient investigation of biopsychosocial determinants was performed in these birth cohorts. For example, the sense of coherence, which is proposed to be an important psychological factor that enables people to cope with stressors and maintain and improve their oral health, was not studied.^
53,86
^ Parental self-efficacy seems to have a relationship to 0- to 5-year-old children’s oral hygiene behaviour. According to Bandura’s concept, self-efficacy is an individual’s confidence in their ability to perform a behaviour.^
66,98
^ The link between self-efficacy and oral health has not been studied. These studies did not attempt to identify the salutary factors that actively promote children’s oral health.^
55
^ No results were reported in relation to oral health literacy, salutogenic factors such as parental locus of control, or oral health literacy, although these have been validated in the literature as determinants of children’s oral health.^
26,93,125
^


Moreover, imprecision remains concerning risk factors. For example, the duration of breastfeeding is a protective factor against caries up to 6 months.^
19,48,62,68,70,74
^ Breastfeeding is a risk factor from age 2.^
16,17,34,76,82,102,106
^ Despite the fact that caries is a multifactorial disease, and that many factors have been taken into account, it is difficult to determine the role of breastfeeding between 6 and 24 months of age. In addition, it should be noted that these results are not in accordance with WHO recommendations. The World Health Organization (WHO) recommends breastfeeding frequently and on demand until the child is two years old or beyond. For infants between six and eight months of age, it is recommended that they consume two to three meals per day. For infants between 9 and 23 months of age, it is recommended that they eat three to four meals a day, with one to two additional snacks as needed.^
126
^ However, the act of snacking and drinking may serve as a confounding factor and could partially explain the observed differences in caries experience between children aged 0 to 5.^20,33,45,48,62,67–70^


Studying pre-school children, this scoping review supports the role of non-nutritive sucking in the development of malocclusions. Between the ages of 0 and 5, most children suffer from non-nutritive sucking. The prevalence of non-nutritive sucking decreases with age.^
12
^


Peres et al^
81
^ present a comprehensive overview of the general characteristics of oral health-related birth cohort studies with two or more oral health publications. The authors also provide a detailed analysis of the design characteristics of these studies, including the specific dental and oral-related measurements, the level of investigation, and the potential investigations in these studies.^
81
^ Peres et al^
81
^ do not present any results from these publications. This scoping review represents a comprehensive addition to the existing literature, with new data for a specific population: children aged 0 to 5 years old.

## Conclusion

Birth cohorts provide an opportunity to explain the origins of children’s oral health disorders between the ages of 0 and 5. This scoping review showed that children’s oral health between the ages of 0 and 5 within birth cohorts is widely studied through caries and the associated risk factors in primary teeth. Early caries childhood is highlighted as a measure of the child’s oral health. However, there has been insufficient investigation of biopsychosocial determinants in these birth cohorts. In addition, the role of the parents, their oral health literacy, and their empowerment with regard to their child’s oral health remains to be investigated.

## References

**Table d67e2886:** 

Biomedical literature database	Query equation
PubMed	(“oral hygiene”[Mesh] OR “oral health”[Mesh] OR “oral hygiene”[TW] OR “oral health”[TW] OR “Dental Care for Children”[Mesh] OR “dental car*”[TW] OR “dental health services”[TW] OR “tooth diseases”[Mesh] OR “dental caries”[TW] OR “tooth disease”[TW] OR “tooth dec*”[TW] OR “home dental care”[TW] OR “dental health”[TW]) AND ((“Longitudinal Studies”[Mesh] OR “birth cohort*”[TW] OR “pediatric cohort*”[TW] OR “cohort analys*”[TW] OR “longitudinal stud*”[TW] OR “longitudinal survey*”[TW] OR “longitudinal evaluation*”[TW]) OR “pelotas birth cohort”[TW] OR “flemish environment and health study”[TW] OR “copenhagen child cohort”[TW] OR “danish national birth cohort”[TW] OR “odense child cohort”[TW] OR “northern finland birth cohort”[TW] OR “elfe”[TW] OR “babycare”[TIAB] OR “giniplus”[TW] OR “lisa plus”[TW] OR “rhea cohort” OR “cork baseline birth cohort study”[TW] OR “Lifeways Cross-Generation Cohort Study”[TW] OR “piccolipiu”[TW] OR “kaunas cohort”[TW] OR “ABCD-study cohort”[TW] OR “gecko drenthe”[TW] OR “generation r study”[TW] OR “koala birth cohort study”[TW] OR “mefab”[TW] OR “piama birth cohort”[TW] OR “pride study”[TW] OR “whistler birth cohort”[TW] OR “arcrisk”[TW] OR “norwegian human milk study”[TW] OR “norwegian mother and child cohort”[TW] OR “moba”[TW] OR “krakow cohort”[TW] OR “inma project”[TW] OR “bamse”[TW] OR “inuendo”[TW] OR “avon longitudinal study of parents and children alspac”[TW] OR “born in bradford”[TW] OR “ehl”[TW] OR “growing up in singapore”[TW] OR “millennium cohort”[TW] OR “dunedin multidisciplinary”[TW] OR “SMILE study” [TW] OR “Jena “ [TW] OR “Hong Kong Children” [TW] OR “ PROMISE-EBF study” [TW] OR “IFS”[TW] OR “ Iowa Fluoride Study” [TW]) AND (“english”[Language]) AND (newborn[Filter] OR allinfant[Filter] OR infant[Filter] OR preschoolchild[Filter] OR “Infant”[Mesh] OR “Child, Preschool”[Mesh] OR “infant*”[TW] OR “preschool*”[TW] OR “newborn*”[TW] OR “toddler*”[TW])
Embase	(‘dental car*’:ti,ab,kw OR ‘mouth hygiene’:ti,ab,kw OR ‘mouth diseas*’:ti,ab,kw OR ‘dental procedure’:ti,ab,kw OR ‘tooth disease’:ti,ab,kw OR ‘oral health’:ti,ab,kw OR ‘pediatric dentis*’:ti,ab,kw OR ‘dental health services’:ti,ab,kw OR ‘home dental care’:ti,ab,kw) AND ((‘birth cohort*’:ti,ab,kw OR ‘pediatric cohort*’:ti,ab,kw OR ‘cohort analys*’:ti,ab,kw OR ‘longitudinal stud*’:ti,ab,kw OR ‘longitudinal survey*’:ti,ab,kw OR ‘longitudinal evaluation*’:ti,ab,kw OR ‘longitudinal study’/de) OR (‘pelotas birth cohort’:ti,ab,kw OR ‘flemish environment and health study’:ti,ab,kw OR ‘copenhagen child cohort’:ti,ab,kw OR ‘danish national birth cohort’:ti,ab,kw OR ‘odense child cohort’:ti,ab,kw OR ‘northern finland birth cohort’:ti,ab,kw OR ‘elfe’:ti,ab,kw OR ‘babycare’:ti,ab OR ‘giniplus’:ti,ab,kw OR ‘lisa plus’:ti,ab,kw OR ‘rhea cohort’ OR ‘cork baseline birth cohort study’:ti,ab,kw OR ‘Lifeways Cross-Generation Cohort Study’:ti,ab,kw OR ‘piccolipiu’:ti,ab,kw OR ‘kaunas cohort’:ti,ab,kw OR ‘ABCD-study cohort’:ti,ab,kw OR ‘gecko drenthe’:ti,ab,kw OR ‘generation r study’:ti,ab,kw OR ‘koala birth cohort study’:ti,ab,kw OR ‘mefab’:ti,ab,kw OR ‘piama birth cohort’:ti,ab,kw OR ‘pride study’:ti,ab,kw OR ‘whistler birth cohort’:ti,ab,kw OR ‘arcrisk’:ti,ab,kw OR ‘norwegian human milk study’:ti,ab,kw OR ‘norwegian mother and child cohort’:ti,ab,kw OR ‘moba’:ti,ab,kw OR ‘krakow cohort’:ti,ab,kw OR ‘inma project’:ti,ab,kw OR ‘bamse’:ti,ab,kw OR ‘inuendo’:ti,ab,kw OR ‘avon longitudinal study of parents and children alspac’:ti,ab,kw OR ‘born in bradford’:ti,ab,kw OR ‘ehl’:ti,ab,kw OR ‘growing up in singapore’:ti,ab,kw OR ‘millennium cohort’:ti,ab,kw OR ‘dunedin multidisciplinary’:ti,ab,kw OR ‘SMILE study’:ti,ab,kw OR ‘Jena ‘:ti,ab,kw OR ‘Hong Kong Children’:ti,ab,kw OR ‘ PROMISE-EBF study’:ti,ab,kw OR ‘IFS’:ti,ab,kw OR ‘ Iowa Fluoride Study’:ti,ab,kw)) AND ([infant]/lim OR [newborn]/lim OR [preschool]/lim OR ‘preschool’:ti,ab,kw OR ‘infant’:ti,ab,kw OR ‘newborn’:ti,ab,kw OR ‘toddler’:ti,ab,kw OR ‘preschool child’/exp OR ‘infant’/exp OR ‘toddler’/exp) AND ([english]/lim)
Scopus	(TITLE-ABS-KEY (“oral hygiene”) OR TITLE-ABS-KEY (“oral health”) OR TITLE-ABS-KEY (“Dental Care for Children”) OR TITLE-ABS-KEY (“dental car*”) OR TITLE-ABS-KEY (“dental health service*”) OR TITLE-ABS-KEY (“tooth diseases”) OR TITLE-ABS-KEY (“dental caries”) OR TITLE-ABS-KEY (“tooth disease”) OR TITLE-ABS-KEY (“tooth dec*”) OR TITLE-ABS-KEY (“home dental care”) OR TITLE-ABS-KEY (“dental health”)) AND ((TITLE-ABS-KEY (“Longitudinal Studies”) OR TITLE-ABS-KEY (“birth cohort*”) OR TITLE-ABS-KEY (“pediatric cohort*”) OR TITLE-ABS-KEY (“cohort analys*”) OR TITLE-ABS-KEY (“longitudinal stud*”) OR TITLE-ABS-KEY (“longitudinal survey*”) OR TITLE-ABS-KEY (“longitudinal evaluation*”)) OR (TITLE-ABS-KEY (“pelotas birth cohort”) OR TITLE-ABS-KEY (“flemish environment and health study”) OR TITLE-ABS-KEY (“copenhagen child cohort”) OR TITLE-ABS-KEY (“danish national birth cohort”) OR TITLE-ABS-KEY (“odense child cohort”) OR TITLE-ABS-KEY (“northern finland birth cohort”) OR TITLE-ABS-KEY (“elfe”) OR TITLE-ABS-KEY (“babycare”) OR TITLE-ABS-KEY (“giniplus”) OR TITLE-ABS-KEY (“lisa plus”) OR TITLE-ABS-KEY (“rhea cohort”) OR TITLE-ABS-KEY (“cork baseline birth cohort study”) OR TITLE-ABS-KEY (“Lifeways Cross-Generation Cohort Study”) OR TITLE-ABS-KEY (“piccolipiu”) OR TITLE-ABS-KEY (“kaunas cohort”) OR TITLE-ABS-KEY (“ABCD-study cohort”) OR TITLE-ABS-KEY (“gecko drenthe”) OR TITLE-ABS-KEY (“generation r study”) OR TITLE-ABS-KEY (“koala birth cohort study”) OR TITLE-ABS-KEY (“mefab”) OR TITLE-ABS-KEY (“piama birth cohort”) OR TITLE-ABS-KEY (“pride study”) OR TITLE-ABS-KEY (“whistler birth cohort”) OR TITLE-ABS-KEY (“arcrisk”) OR TITLE-ABS-KEY (“norwegian human milk study”) OR TITLE-ABS-KEY (“norwegian mother and child cohort”) OR TITLE-ABS-KEY (“moba”) OR TITLE-ABS-KEY (“krakow cohort”) OR TITLE-ABS-KEY (“inma project”) OR TITLE-ABS-KEY (“bamse”) OR TITLE-ABS-KEY (“inuendo”) OR TITLE-ABS-KEY (“avon longitudinal study of parents and children alspac”) OR TITLE-ABS-KEY (“born in bradford”) OR TITLE-ABS-KEY (“ehl”) OR TITLE-ABS-KEY (“growing up in singapore”) OR TITLE-ABS-KEY (“millennium cohort”) OR TITLE-ABS-KEY (“dunedin multidisciplinary”) OR TITLE-ABS-KEY (“SMILE study” ) OR TITLE-ABS-KEY (“Jena “) OR TITLE-ABS-KEY (“Hong Kong Children” ) OR TITLE-ABS-KEY (“ PROMISE-EBF study” ) OR TITLE-ABS-KEY (“IFS”) OR TITLE-ABS-KEY (“Iowa Fluoride Study”))) AND (TITLE-ABS-KEY (“preschool*”) OR TITLE-ABS-KEY (“newborn*”) OR TITLE-ABS-KEY (“toddler*”) OR TITLE-ABS-KEY (“infant*”)) AND ( ( LANGUAGE ( english )) )
Web of Science	((((TS=(“oral hygiene” OR “oral health” OR “Dental Care for Children” OR “dental car*” OR “dental health services” OR “tooth diseases” OR “dental caries” OR “tooth disease” OR “tooth dec*” OR “home dental care” OR “dental health”)) OR TI=(“oral hygiene” OR “oral health” OR “Dental Care for Children” OR “dental car*” OR “dental health services” OR “tooth diseases” OR “dental caries” OR “tooth disease” OR “tooth dec*” OR “home dental care” OR “dental health”)) OR AB=(“oral hygiene” OR “oral health” OR “Dental Care for Children” OR “dental car*” OR “dental health services” OR “tooth diseases” OR “dental caries” OR “tooth disease” OR “tooth dec*” OR “home dental care” OR “dental health”)) OR AK=(“oral hygiene” OR “oral health” OR “Dental Care for Children” OR “dental car*” OR “dental health services” OR “tooth diseases” OR “dental caries” OR “tooth disease” OR “tooth dec*” OR “home dental care” OR “dental health”)) AND KP=(“oral hygiene” OR “oral health” OR “Dental Care for Children” OR “dental car*” OR “dental health services” OR “tooth diseases” OR “dental caries” OR “tooth disease” OR “tooth dec*” OR “home dental care” OR “dental health”) AND (((((TS=(«Longitudinal Studies» OR «birth cohort*» OR «pediatric cohort*» OR «cohort analys*» OR «longitudinal stud*» OR «longitudinal survey*» OR «longitudinal evaluation*»)) OR TI=(«Longitudinal Studies» OR «birth cohort*» OR «pediatric cohort*» OR «cohort analys*» OR «longitudinal stud*» OR «longitudinal survey*» OR «longitudinal evaluation*»)) OR AB=(«Longitudinal Studies» OR «birth cohort*» OR «pediatric cohort*» OR «cohort analys*» OR «longitudinal stud*» OR «longitudinal survey*» OR «longitudinal evaluation*»)) OR AK=(«Longitudinal Studies» OR «birth cohort*» OR «pediatric cohort*» OR «cohort analys*» OR «longitudinal stud*» OR «longitudinal survey*» OR «longitudinal evaluation*»)) AND KP=(«Longitudinal Studies» OR «birth cohort*» OR «pediatric cohort*» OR «cohort analys*» OR «longitudinal stud*» OR «longitudinal survey*» OR «longitudinal evaluation*») OR ((((TS=(“pelotas birth cohort” OR “flemish environment and health study” OR “copenhagen child cohort” OR “danish national birth cohort” OR “odense child cohort” OR “northern finland birth cohort” OR “elfe” OR “babycare” OR “giniplus” OR “lisa plus” OR “rhea cohort” OR “cork baseline birth cohort study” OR “Lifeways Cross-Generation Cohort Study” OR “picolinic” OR “kaunas cohort” OR “ABCD-study cohort” OR “gecko drenthe” OR “generation r study” OR “koala birth cohort study” OR “metab” OR “piama birth cohort” OR “pride study” OR “whistler birth cohort” OR “arcwise” OR “norwegian human milk study” OR “norwegian mother and child cohort” OR “moby” OR “krakow cohort” OR “inma project” OR “basse” OR “innuendos” OR “avon longitudinal study of parents and children alspac” OR “born in bradford” OR “ehl” OR “growing up in singapore” OR “millennium cohort” OR “dunedin multidisciplinary” OR “SMILE study” OR “Jena “ OR “Hong Kong Children”OR “PROMISE-EBF study” OR “IFS” OR “ Iowa Fluoride Study”)) OR TI=(“pelotas birth cohort” OR “flemish environment and health study” OR “copenhagen child cohort” OR “danish national birth cohort” OR “odense child cohort” OR “northern finland birth cohort” OR “elfe” OR “babycare” OR “giniplus” OR “lisa plus” OR “rhea cohort” OR “cork baseline birth cohort study” OR “Lifeways Cross-Generation Cohort Study” OR “picolinic” OR “kaunas cohort” OR “ABCD-study cohort” OR “gecko drenthe” OR “generation r study” OR “koala birth cohort study” OR “metab” OR “piama birth cohort” OR “pride study” OR “whistler birth cohort” OR “arcwise” OR “norwegian human milk study” OR “norwegian mother and child cohort” OR “moby” OR “krakow cohort” OR “inma project” OR “basse” OR “innuendos” OR “avon longitudinal study of parents and children alspac” OR “born in bradford” OR “ehl” OR “growing up in singapore” OR “millennium cohort” OR “dunedin multidisciplinary” OR “SMILE study” OR “Jena “ OR “Hong Kong Children”OR “PROMISE-EBF study” OR “IFS” OR “ Iowa Fluoride Study”)) OR AB=(“pelotas birth cohort” OR “flemish environment and health study” OR “copenhagen child cohort” OR “danish national birth cohort” OR “odense child cohort” OR “northern finland birth cohort” OR “elfe” OR “babycare” OR “giniplus” OR “lisa plus” OR “rhea cohort” OR “cork baseline birth cohort study” OR “Lifeways Cross-Generation Cohort Study” OR “picolinic” OR “kaunas cohort” OR “ABCD-study cohort” OR “gecko drenthe” OR “generation r study” OR “koala birth cohort study” OR “metab” OR “piama birth cohort” OR “pride study” OR “whistler birth cohort” OR “arcwise” OR “norwegian human milk study” OR “norwegian mother and child cohort” OR “moby” OR “krakow cohort” OR “inma project” OR “basse” OR “innuendos” OR “avon longitudinal study of parents and children alspac” OR “born in bradford” OR “ehl” OR “growing up in singapore” OR “millennium cohort” OR “dunedin multidisciplinary” OR “SMILE study” OR “Jena “ OR “Hong Kong Children”OR “PROMISE-EBF study” OR “IFS” OR “ Iowa Fluoride Study”)) OR AK=(“pelotas birth cohort” OR “flemish environment and health study” OR “copenhagen child cohort” OR “danish national birth cohort” OR “odense child cohort” OR “northern finland birth cohort” OR “elfe” OR “babycare” OR “giniplus” OR “lisa plus” OR “rhea cohort” OR “cork baseline birth cohort study” OR “Lifeways Cross-Generation Cohort Study” OR “picolinic” OR “kaunas cohort” OR “ABCD-study cohort” OR “gecko drenthe” OR “generation r study” OR “koala birth cohort study” OR “metab” OR “piama birth cohort” OR “pride study” OR “whistler birth cohort” OR “arcwise” OR “norwegian human milk study” OR “norwegian mother and child cohort” OR “moby” OR “krakow cohort” OR “inma project” OR “basse” OR “innuendos” OR “avon longitudinal study of parents and children alspac” OR “born in bradford” OR “ehl” OR “growing up in singapore” OR “millennium cohort” OR “dunedin multidisciplinary” OR “SMILE study” OR “Jena “ OR “Hong Kong Children”OR “PROMISE-EBF study” OR “IFS” OR “ Iowa Fluoride Study”)) AND KP=(“pelotas birth cohort” OR “flemish environment and health study” OR “copenhagen child cohort” OR “danish national birth cohort” OR “odense child cohort” OR “northern finland birth cohort” OR “elfe” OR “babycare” OR “giniplus” OR “lisa plus” OR “rhea cohort” OR “cork baseline birth cohort study” OR “Lifeways Cross-Generation Cohort Study” OR “picolinic” OR “kaunas cohort” OR “ABCD-study cohort” OR “gecko drenthe” OR “generation r study” OR “koala birth cohort study” OR “metab” OR “piama birth cohort” OR “pride study” OR “whistler birth cohort” OR “arcwise” OR “norwegian human milk study” OR “norwegian mother and child cohort” OR “moby” OR “krakow cohort” OR “inma project” OR “basse” OR “innuendos” OR “avon longitudinal study of parents and children alspac” OR “born in bradford” OR “ehl” OR “growing up in singapore” OR “millennium cohort” OR “dunedin multidisciplinary” OR “SMILE study” OR “Jena “ OR “Hong Kong Children”OR “PROMISE-EBF study” OR “IFS” OR “ Iowa Fluoride Study”)) AND ((((TS=(“infant*” OR “preschool*” OR “newborn*” OR “toddler*”)) OR TI=(“infant*” OR “preschool*” OR “newborn*” OR “toddler*”)) OR AB=(“infant*” OR “preschool*” OR “newborn*” OR “toddler*”)) OR AK=(“infant*” OR “preschool*” OR “newborn*” OR “toddler*”)) AND KP=(“infant*” OR “preschool*” OR “newborn*” OR “toddler*”)
DOSS	(DE (Infant dental care OR Children’s dental care OR pediatric dentistry OR Pediatric oral medicine OR Dental caries in children) AB (dental caries OR mouth hygiene OR mouth disease* OR dental procedure* OR dental health services OR tooth disease* OR dental care OR pediatric dentistry OR oral health OR oral hygiene) OR TI (dental caries OR mouth hygiene OR mouth disease* OR dental health services OR dental procedure* OR tooth disease* OR dental care OR pediatric dentistry OR oral health OR oral hygiene) OR KW (dental caries OR mouth hygiene OR mouth disease* OR dental procedure* OR tooth disease* OR dental care OR pediatric dentistry OR dental health services OR oral health OR oral hygiene OR Infant dental care OR Children’s dental care OR pediatric dentistry OR Pediatric oral medicine OR Dental caries in children)) AND (DE (longitudinal method) OR AB (longitudinal stud* OR longitudinal evaluation* OR longitudinal survey* OR birth cohort* OR pediatric cohort* OR cohort analys*) OR TI (longitudinal stud* OR longitudinal evaluation* OR longitudinal survey* OR birth cohort* OR pediatric cohort* OR cohort analys*) OR KW (longitudinal stud* OR longitudinal evaluation* OR longitudinal survey* OR birth cohort* OR pediatric cohort* OR cohort analys*)) AND (DE (Infants OR Toddlers OR Preschool children) OR AB (newborn OR infant* OR preschool OR toddler*) OR TI (newborn OR infant* OR preschool OR toddler*) OR KW (newborn OR infant* OR preschool OR toddler*)) AND LA (English)
	
